# On the Zwitterionic Nature of Gas-Phase Peptides and Protein Ions

**DOI:** 10.1371/journal.pcbi.1000775

**Published:** 2010-05-06

**Authors:** Roberto Marchese, Rita Grandori, Paolo Carloni, Simone Raugei

**Affiliations:** 1 Statistical and Biological Physics Sector, International School for Advanced Studies (SISSA-ISAS) and DEMOCRITOS, Trieste, Italy; 2 Department of Bioscience and Biotechnology, Milano-Bicocca, Milano, Italy; 3 Italian Institute of Technology (IIT), Trieste, Italy; 4 Computational Biophysics sector, German Research School for Simulation Science, FZ-Juelich and RWTH, Juelich, Germany; 5 Center for Molecular Electrocatalysis, Pacific Northwest National Laboratory, Richland, Washington, United States of America; University of Houston, United States of America

## Abstract

Determining the total number of charged residues corresponding to a given value of net charge for peptides and proteins in gas phase is crucial for the interpretation of mass-spectrometry data, yet it is far from being understood. Here we show that a novel computational protocol based on force field and massive density functional calculations is able to reproduce the experimental facets of well investigated systems, such as angiotensin II, bradykinin, and tryptophan-cage. The protocol takes into account all of the possible protomers compatible with a given charge state. Our calculations predict that the low charge states are zwitterions, because the stabilization due to intramolecular hydrogen bonding and salt-bridges can compensate for the thermodynamic penalty deriving from deprotonation of acid residues. In contrast, high charge states may or may not be zwitterions because internal solvation might not compensate for the energy cost of charge separation.

## Author Summary

In the last two decades mass spectrometry has given an impressive contribution to biochemistry, protein science, proteomics and structural biology. This technique offers powerful insights into protein structure and dynamics along with useful information on the role of solvent in protein stability as it is able to preserve non-covalent interactions and globular structures during the proteins' flight inside the mass spectrometer. Unfortunately, the key issue of the charge state of ionizable groups, presumably different from that in solution, has not been elucidated yet. So far conflicting assumptions and conclusions have been drawn by several groups. In the present work a very accurate structural and energetic analysis of the protonation state of two peptides and a small protein in the gas phase was performed. Results suggest that internal solvation can stabilize charge separation with the formation of zwitterionic states.

## Introduction

Predicting the structural properties of proteins in the gas phase is crucial to interpret mass spectrometry data, yet this is far from being understood [1]–[10]. So far, it has been established that (*i*) compact structures acquire smaller net charges than unfolded ones [11]–[13], (*ii*) secondary and tertiary structure elements play a crucial role for protein fragmentation [14]–[23], and (*iii*) hydrogen bonds (H-bonds) and salt-bridges [3], [24], [25] may stabilize the structures. However, how desolvation impacts on structural facets of proteins [2], [3], [8], [26]–[30], peptides [3], [31]–[35] and even single amino acids [36]–[45] is matter of a vivid debate.

A key point is the presence of charge separation. Whilst amino acids exist mostly in their zwitterionic form in the aqueous solution [31], [36], [40], [46], conflicting assumptions and conclusions have been drawn for the same molecules *in vacuo* [47]–[50]. For peptides and proteins, the key issue of the charge state of ionizable groups, presumably different from that in solution, is even less clear [2], [51]–[53]. One line of thought assumes neutral acidic functions for proteins analyzed in positive-ion mode (*i.e.*, generating and detecting positively charged ions) and neutral basic sites in negative-ion mode. In other words, the number of ionized groups is generally assumed to be equal to the net charge of the protein ion [54]–[56]. Electrostatic energy calculations based on this supposition fail to reproduce experimental values of apparent gas-phase basicity (GPB) for folded protein ions [57]. The GPB of a basic species B is defined as the negative of the free-energy change, 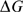, for the gas-phase protonation reaction
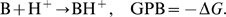
If B is the conjugate base of an acid AH, then 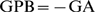, where GA is the gas-phase acidity of AH. Analogously, the proton affinity is defined as the negative of the protonation enthalpy, PA = −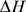.

In contrast, an increasing number of experimental [16], [24], [25], [58]–[61] and theoretical [62], [63] investigations carried out on peptides and small proteins indicate that zwitterionic states may survive in the absence of solvent if the structural features allow for adequate intramolecular solvation [64]–[67]. Recent ultraviolet photo-dissociation [16] and fluorescence [25], [61] experiments indicate the presence of stabilizing salt-bridge motifs in small biomolecules. Salt bridges exist also in protonated, gas-phase serine dimers [24] and have been predicted for arginine dimers [63], [68], [69]. These interactions add to other stabilizing contributions such as hydrogen bonds [3], [24], [25]. Molecular dynamics (MD) simulations on a minimalistic lattice model of a zwitterionic system [1] turned out to reproduce the experimental observation that compact structures acquire smaller net charges than unfolded ones [11]–[13]. On the basis of these simulations, it has been also proposed that steric and electrostatic shielding of charged residues in compact conformations are the major factors responsible for this structural facet. Energy calculations [2], [7], [52] and measurements [51], [70] on several well characterized proteins in their experimentally observed, most populated charge state suggest that the presence of zwitterions depends on the specific protein structure [2], [51]. Deprotonated aspartic and glutamic residues persist in the most abundant, positively charged protomer of insulin, the C-terminal domain of the ribosomal protein L7/L12 and ubiquitin, but not in tryptophan-cage and lysozyme [2].

Prompted by the current lack of understanding of the charge state of protein ions *in vacuo*, here we have carried out an exhaustive energy analysis on three systems largely studied in the gas phase both experimentally [16], [25], [59], [60], [71]–[77] and theoretically [52], [62], [63], [72]. These are the 8-residue peptide angiotensin II (AN) [74]–[76] and the 9-residue peptide bradykinin (BK) [16], [59], [62], [63], [71]–[73], as well as the tryptophan-cage (Trp-cage) [16], [25], [52], [60], [77] protein. The latter is a 20-residue mini-protein with a well defined secondary and tertiary structure in aqueous solution at ambient conditions. It consists of an 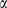-helix and a compact hydrophobic core formed by a Trp side chain from the 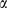-helix, surrounded by several hydrophobic residues (two prolines and one tyrosine) [78].

A complete exploration of the protomer space (*i.e.*, all of the possible charge configurations compatible with a given charge state) of these biomolecules is performed coupling force field–based molecular dynamics and density functional theory (DFT) calculations. In contrast to previous computational studies [25], [31], [52], [62], [63], [79], [80], all of the charge states generated by ionized and/or neutral *basic* (R, K, H, Q, N-terminus) and *acidic* groups (E, D, C-terminus), featuring more than one protomer, are taken into account.

A computational protocol apt to this task has been developed, allowing for an exhaustive exploration of the conformational space of each protomer. Based on such protocol, we suggest that low-charge states are likely zwitterions. In those cases, H-bonds and salt-bridges stabilize largely zwitterionic states, considerably reducing the differences in the apparent GPB between basic residues and the conjugated base of acidic residues. At high net charge, instead, non-zwitterion states are most likely.

## Methods

### Systems

The sequences of BK, AN and Trp-cage are RPPGFSPFR, DRVYIHPF, NLYIQWLKDGGPSSGRPPPS, respectively. For each system, the following protonation sites were considered: 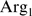, 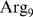, N- and C-term for BK; 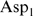, 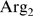, 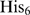, N- and C-term for AN (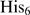 in the neutral state can be protonated either in 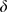 or 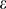, both tautomers were considered); 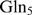, 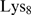, 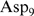, 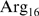, and N- and C-term for Trp-cage. In the latter, protonation of 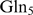 was considered for the 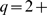 and 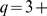 charge states on the basis of experimental evidences [19].

BK and AN have no determined secondary structure and all of the calculations started with an all-trans backbone and side-chain conformation. Instead, the Trp-cage initial structure was obtained by a 20-ns MD simulation in aqueous solution at ambient conditions based on the NMR structure number 1 deposited in the protein data bank (PDB code: 1L2Y) [78] (see Text S1). The most probable protonation state in water [78] was chosen.

For the chosen set of protonation sites, all of the charge states which feature more than one protomer were taken into account. For these charge states, all of the possible protomers were considered, for a total of 100 protomers (see Tables 1, 2, and 3).

**Table 1:** Energetics and structural parameters for the lowest-energy conformers of bradykinin protomers.

N-ter	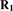	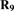	C-ter	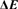	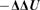	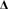 PA	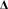 GB	IR	SB	sHB	iHB	HB
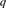 = 0												
0	+	0	−	0	380	409	368	2	1	2	5	1
0	0	+	−	10	380	409	368	2	1	2	4	3
+	0	0	−	49	542	535	540	2	1	1	5	0
0	0	0	0	57	0	0	0	0	0	0	0	4
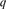 = 1+												
0	+	+	−	0	380	409	368	3	2	3	2	0
0	+	0	0	46	0	0	0	1	0	0	3	4
0	0	+	0	63	0	0	0	1	0	0	4	1
+	0	+	−	67	542	535	540	3	2	2	2	3
+	+	0	−	112	542	535	540	3	1	2	4	0
+	0	0	0	152	162	126	172	1	0	0	3	3
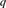 = 2+												
+	+	+	−	0	542	535	540	4	3	4	3	1
0	+	+	0	77	0	0	0	2	0	0	6	1
+	+	0	0	85	162	126	172	2	0	0	5	1
+	0	+	0	93	162	126	172	2	0	0	5	2

In each row the following information is reported: protonation pattern (first column); energy difference with respect to the most stable protomer (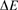 in kJ/mol); (intrinsic) internal energy variation (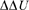), proton affinity (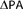
PA) and gas-phase basicity (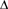
GPB) relative to the most favourable protomer (see text for a definition of these quantities; all values are in kJ/mol); ionized residues (IR); salt-bridges (SB); hydrogen bonds between salt-bridged residues (sHB); ionized hydrogen bonds where either the donor or the acceptor is ionized, 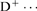
A or D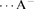 H-bonds (iHB); neutral hydrogen bonds (HB). Hydrogen bonds are identified according to the donor-acceptor (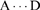) distance and the donor-acceptor H-bond angle (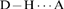). The following geometric criterion was adopted: 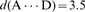
Å and 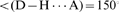. A salt-bridge is formed if the distance between any oxygen atom of the acidic residue and any protonable nitrogen atom of the basic residue is less than 4.0 Å.

**Table 2:** Energetics and structural parameters for the lowest-energy conformers of angiotensin II protomers.

N-ter	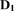	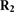	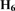	C-ter	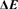	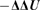	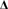 PA	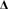 GB	IR	SB	sHB	iHB	HB
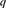 = 1−													
0	−	+	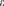	−	0	380	409	368	3	2	4	2	1
0	−	+	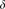	−	43	380	409	368	3	2	4	2	1
0	−	0	+	−	51	447	452	423	3	2	2	2	1
0	0	0	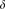	−	54	0	0	0	1	0	0	6	2
0	−	0	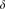	0	73	95	105	66	1	0	0	5	2
+	−	0	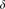	−	84	542	535	540	3	2	1	6	0
0	−	0	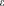	0	86	95	105	66	1	0	0	4	1
0	0	0	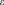	−	91	0	0	0	1	0	0	3	4
+	−	0	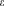	−	100	542	535	540	3	2	1	5	0
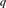 = 0													
0	−	+	+	−	0	732	756	725	4	4	5	0	0
0	0	+	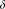	−	17	380	409	368	2	1	1	3	2
0	0	+	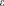	−	21	380	409	368	2	1	2	1	2
0	0	0	+	−	32	447	452	423	2	0	1	4	0
0	−	+	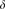	0	37	285	304	302	2	1	2	3	1
+	−	+	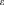	−	39	827	839	842	4	4	5	2	4
+	−	+	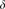	−	39	827	839	842	4	4	4	1	2
0	−	+	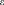	0	42	285	304	302	2	1	2	3	4
+	0	0	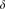	−	64	542	535	540	2	1	1	2	0
0	0	0	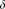	0	69	0	0	0	0	0	0	0	7
+	0	0	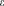	−	73	542	535	540	2	1	1	3	4
0	−	0	+	0	80	352	347	357	2	1	1	3	3
+	−	0	+	−	100	894	882	897	4	4	3	2	0
+	−	0	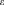	0	104	447	430	474	2	1	0	4	2
0	0	0	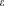	0	105	0	0	0	0	0	0	0	8
+	−	0	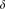	0	106	447	430	474	2	1	1	2	2
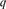 = 1+													
0	0	+	+	−	0	447	452	423	3	2	3	3	1
+	−	+	+	−	1	894	882	897	5	4	5	2	0
0	−	+	+	0	2	352	347	357	3	2	3	3	0
0	0	+	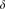	0	11	0	0	0	1	0	0	4	4
0	0	+	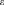	0	25	0	0	0	1	0	0	4	2
+	−	+	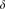	0	41	447	430	474	3	2	2	3	0
0	0	0	+	0	42	67	43	55	1	0	0	2	3
+	0	+	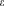	−	48	534	542	585	3	2	2	4	1
+	−	+	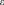	0	48	542	535	540	3	2	2	2	1
+	0	+	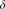	−	62	542	535	540	3	2	2	2	1
+	0	0	+	−	81	447	430	474	3	2	2	3	1
+	0	0	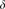	0	85	162	126	172	1	0	0	3	1
+	−	0	+	0	117	514	473	529	3	2	2	1	2
+	0	0	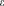	0	122	162	126	172	1	0	0	2	3
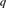 = 2+													
0	0	+	+	0	0	0	0	0	2	0	0	4	1
+	0	+	+	−	15	542	535	540	4	3	3	4	1
+	0	+	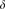	0	19	95	83	117	2	0	0	2	3
+	−	+	+	0	43	447	430	474	4	3	3	2	0
+	0	0	+	0	64	162	126	172	2	0	0	2	1
+	0	+	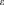	0	95	95	83	117	2	0	0	2	2

In each row the following information is reported: protonation pattern (first column); energy difference with respect to the most stable protomer (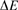 in kJ/mol); (intrinsic) internal energy variation (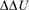), proton affinity (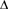
PA) and gas-phase basicity (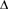
GPB) relative to the most favourable protomer (see text for a definition of these quantities; all values are in kJ/mol); ionized residues (IR); salt-bridges (SB); hydrogen bonds between salt-bridged residues (sHB); ionized hydrogen bonds where either the donor or the acceptor is ionized, 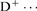
A or D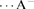 H-bonds (iHB); neutral hydrogen bonds (HB). Hydrogen bonds are identified according to the donor-acceptor (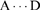) distance and the donor-acceptor H-bond angle (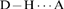). The following geometric criterion was adopted: 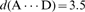
Å and 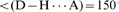. A salt-bridge is formed if the distance between any oxygen atom of the acidic residue and any protonable nitrogen atom of the basic residue is less than 4.0 Å.

**Table 3:** Energetics and structural parameters for the lowest-energy conformers of Trp-cage protomers.

N-ter	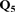	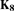	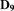	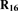	C-ter	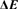	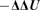	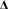 PA	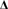 GB	IR	SB	sHB	iHB	HB
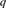 = 0														
0	0	0	0	+	−	0	380	409	368	2	0	0	9	8
+	0	0	−	+	−	9	827	839	842	4	4	4	8	7
0	0	+	−	0	0	20	341	345	350	2	1	1	5	12
0	0	0	0	0	0	22	0	0	0	0	0	0	0	19
0	0	+	0	0	−	25	436	450	416	2	0	0	6	11
0	0	+	−	+	−	33	721	754	718	4	2	3	10	5
0	0	0	−	+	0	47	285	304	302	2	1	1	6	7
+	0	+	−	0	−	95	883	880	890	4	2	2	8	10
+	0	0	0	0	−	108	542	535	540	2	1	1	3	12
+	0	0	−	0	0	228	447	430	474	2	0	0	8	8
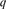 = 1+														
**+**	**0**	**+**	**−**	**+**	**−**	**wat**	**-**	**-**	**-**	**5**	**1**	**1**	**0**	**5**
+	0	0	0	+	−	0	542	535	540	3	1	1	5	12
0	0	0	0	+	0	5	0	0	0	1	0	0	3	12
+	0	0	−	+	0	18	447	430	474	3	2	3	6	6
0	0	+	−	+	0	41	341	345	450	3	2	3	3	11
+	0	0	0	0	0	66	162	126	172	1	0	0	2	16
+	0	+	0	0	−	66	598	576	588	3	1	1	5	8
+	0	+	−	+	−	72	883	880	890	5	5	7	4	5
0	0	+	0	+	−	79	436	450	416	3	0	0	9	7
0	0	+	0	0	0	87	56	41	48	1	0	0	3	13
+	0	+	−	0	0	99	503	471	522	3	1	1	8	7
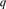 = 2+														
+	0	+	0	+	−	0	542	535	540	4	2	1	6	8
0	0	+	0	+	0	7	0	0	0	2	0	0	6	11
+	0	0	0	+	0	57	106	85	124	2	0	0	6	14
+	+	+	0	0	−	69	674	642	647	4	1	3	7	9
+	+	+	−	+	−	74	959	946	949	6	4	6	7	4
+	0	+	−	+	0	104	447	430	474	4	1	1	11	6
+	0	+	0	0	0	105	162	126	172	2	0	0	4	9
+	+	0	0	+	−	110	618	601	599	4	2	4	5	9
0	+	+	0	+	−	116	512	516	475	4	1	2	6	8
0	+	0	0	+	0	130	76	66	59	2	0	0	7	9
0	+	+	−	+	0	144	417	411	409	4	2	3	9	8
+	+	+	−	0	0	155	579	537	581	4	1	2	8	9
0	+	+	0	0	0	132	107	107	149	2	0	0	4	11
+	+	0	0	0	0	211	238	192	231	2	0	0	6	12
+	+	0	−	+	0	264	523	496	533	4	0	0	12	7
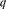 = 3+														
+	0	+	0	+	0	0	30	19	65	3	0	0	10	11
+	+	0	0	+	0	61	106	85	124	3	0	0	8	8
+	+	+	0	+	−	62	542	535	540	5	1	1	12	9
0	+	+	0	+	0	84	0	0	0	3	0	0	8	7
+	+	+	−	+	0	113	447	430	474	5	2	3	9	6
+	+	+	0	0	0	158	162	126	172	3	0	0	7	10

In each row the following information is reported: protonation pattern (first column); energy difference with respect to the most stable protomer (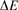 in kJ/mol); (intrinsic) internal energy variation (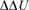), proton affinity (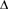
PA) and gas-phase basicity (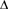
GPB) relative to the most favourable protomer (see text for a definition of these quantities; all values are in kJ/mol); ionized residues (IR); salt-bridges (SB); hydrogen bonds between salt-bridged residues (sHB); ionized hydrogen bonds where either the donor or the acceptor is ionized, 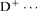
A or D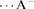 H-bonds (iHB); neutral hydrogen bonds (HB). Hydrogen bonds are identified according to the donor-acceptor (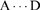) distance and the donor-acceptor H-bond angle (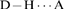). The following geometric criterion was adopted: 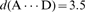
Å and 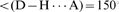. A salt-bridge is formed if the distance between any oxygen atom of the acidic residue and any protonable nitrogen atom of the basic residue is less than 4.0 Å. Structural data for the most stable protonation state in aqueous solution (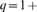) are also reported (data in Italics).

### Force field-based MD calculations

OPLS/AA force field-based [81], [82], constant-temperature MD calculations and geometry optimizations were carried out. The cutoff of electrostatics and van der Waals interactions was fixed at 0.7nm. In the MD simulations, the equations of motion were numerically integrated with a time step of 1.5 fs. All the hydrogen-bond lengths were kept fixed using the LINCS [83] algorithm. The temperature was controlled by the Nosé-Hoover thermostat [84]. The results of force field based MD simulations depend critically on the charge state used. Therefore, we performed a simulation for each protonation state. Specifically 8-ns MD simulations at high-temperature (700K for AN and BK, 350K for Trp-cage) were performed for each protonation state. The chosen temperatures were selected after several careful tests. In particular, for Trp-cage, a temperature of 350K turns out to allow for an exhaustive sampling of the side chains conformations without disrupting, in the relatively short simulation time, the secondary structure. The resulting trajectories were split into 5-ps, non overlapping time windows. For each window, the geometry of the lowest-energy MD conformation was optimized by a conjugated gradient scheme up to 0.1 kJ/molÅ residual force on any atom. This simulated annealing-like procedure yielded for each protomer a large set of conformations. The geometry of structures within 100 kJ/mol (60 kJ/mol for Trp-cage) from the lowest-energy force field conformer were refined at the *ab initio* level (see Section “Identifying relevant protomers of a given charge state”). With this criterion, 60 conformers (35 for Trp-cage), were randomly selected from equally spaced energy windows, one from each window, and re-optimized at DFT/BLYP level of theory.

The GROMACS [85] software package was used for all MD calculations.

### Quantum-chemical geometry optimizations

Quantum-chemical geometry optimizations were performed within the framework of DFT. The Becke exchange [86] and Lee-Yang-Parr [87] correlation functionals (BLYP) were used in a hybrid Gaussian and plane wave approach [88]. The wave function was optimized by using an orbital transformation technique [89] and analytic Goedecker-Teter-Hutter [90], [91] pseudopotentials (PP). The TZV2P Gaussian basis set was used for valence electrons of all atoms, while the auxiliary electron density was expanded in plane waves up to a cutoff of 280 Ry.

The interaction between periodic images in the reciprocal space was removed according to the decoupling scheme presented in [92]. The calculations were carried out with the CP2K code [89], [93], [94], which has been shown to be very efficient for these systems.

The adopted DFT scheme was validated against more accurate (and more expensive) quantum chemistry methods. First, the relative energy of canonical and zwitterionic arginine conformers calculated with the present scheme agrees well with that obtained from all-electrons B3LYP, MP2, and CCSD calculations (see Table 2 in Text S1). Second, all of the 14 protomers of AN with total charge 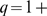 underwent all-electrons, single-point energy evaluations at DFT/B3LYP level with the 6–311++G(d,p) basis set using the Gaussian03 code [95] (Angiotensin II was chosen because it is the smallest of the three molecules studied and, in particular, the charge state 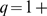 was considered because it presents the largest set of protomers, and it is, therefore, a good benchmark case).These and the previous calculations provided the same energy ranking (see Table 3 in Text S1).

A final concern for using DFT for non-covalent systems is the underestimation of dispersion forces [96], [97]. This flaw of the current GGA functionals might influence the conformational energy, especially in the case of large molecular assemblies like those considered here. To quantify this error an estimate of the dispersion energy was performed for the DFT optimized structures using the OPLS/AA force field. The results of this calculation (see Tables 4, 5 and 6 in Text S1) indicate that the dispersion energy is not expected to change qualitatively the DFT energy ranking of protomers.

## Results

### Identifying relevant protomers of a given charge state

A standard procedure to identify the relevant protomers is currently lacking, even for peptides with more than a few amino-acids. On the one hand, the high complexity of the conformational space hampers an exhaustive search based on first-principle quantum chemistry (such as DFT) of the minimum-energy conformers. On the other hand, force field–based calculations [62], [63], [98], [99], or semiempirical quantum chemical methods [52], may not be accurate enough. For instance, Merck molecular force field [100] energies have been shown to correlate poorly with those calculated at the DFT/B3LYP level [62], [63]. In addition, the energies calculated by force fields do not take into account higher-order effects, which may play a role in our systems. DFT can, instead, take such effects into account.

**Figure 1:**
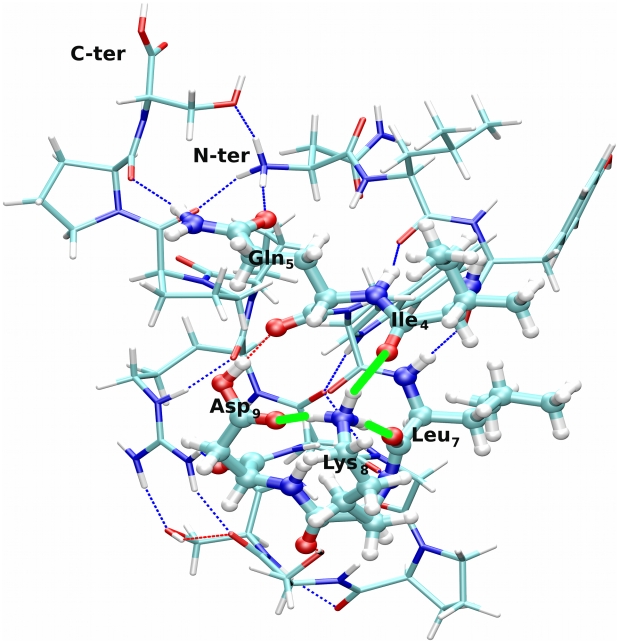
Ball-and-stick representation of the structure of the lowest-energy protomer 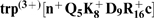 of Trp-cage at 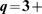. The H-bonding network of protonated 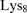 is shown as green lines; other H-bonds are shown as dotted lines.

However, if the empirically calculated conformer is much higher in energy than another (say with a 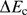 greater than few hundreds of kJ/mol), it will be highly probable that the same ranking holds at the *ab initio* level (see Figure 1 in Text S1). Here, we seek such 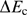 value by performing MD simulations based on the OPLS/AA, which offers the most complete set of base/conjugate acid pairs. The calculations on the three systems *in vacuo* provided several hundreds conformations, which then underwent DFT/BLYP [86], [87] geometry optimizations. Such quantum chemical scheme is extremely efficient for large molecules, as those investigated here [101], [102].

**Figure 2:**
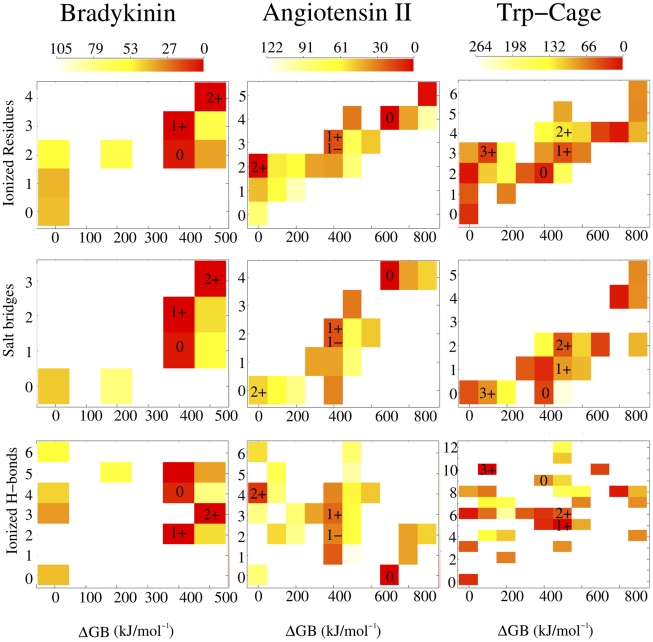
Energy/structure relationship for angiotensin II, bradykinin, and Trp-cage. The color scale refers to the average energy (kJ/mol) of polypeptides with the given pair of parameters. 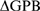 stands for thermodynamic penalty to create a zwitterionic state expressed in terms of residues intrinsic gas-phase basicities (see text for the definition). In each panel, numbers indicate the location of lowest-energy protomers.

We found that less than 5% of the *ab initio* conformers located within 10kJ/mol from the energy minimum fall more than 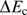 = 100kJ/mol (60kJ/mol for Trp-cage) above the OPLS/AA minimum (see Figure 2 in Text S1). Exploiting this fact, we used the ensuing protocol to identify the lowest-energy minimum for each charge state for each peptide: (*i*) generation of conformers for all possible protomers by OPLS/AA MD and simulated annealing-like calculations; (*ii*) elimination of conformers whose energy is larger than 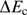 from the absolute minimum; (*iii*) DFT/BLYP geometry optimization of the conformers within 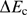; (*iv*) ranking of the conformers based on their DFT energies.

Errors of this protocol are associated with (*i*) the accuracy of the DFT approach, (*ii*) limitations of sampling and ( *iii*) absence of entropy contributions. This points are discussed in the following.

- The accuracy of our DFT-based calculations may be assessed by comparing the energy contributions of the key quantities for the identification of the lowest free-energy minimum with high-level quantum chemical calculations. These are the gas-phase basicities (GPBs) and/or proton affinities (PAs) of the protomers. Table 4, and Tables 1, 2 and 3 in Text S1 show that our approach agrees well with more accurate calculations [62], [103]–[105] (see Section “Quantum-chemical geometry optimizations” for further details). Similar considerations can be evinced from a comparison between our calculations of PA and those recently reported in a highly accurate quantum-chemical study on side chain interactions in proteins [99]. All of these facts establish the accuracy of our calculated energies.
- The upper-bound estimate of the uncertainty in the energy value due to the sampling can be obtained by performing different searches for the lowest-energy conformer of selected protomers. This procedure ended with either the same or very similar structures (backbone root mean square displacements less than 1.0 Å ), and all were located within 10kJ/mol from each other. Therefore, this value can be taken as an estimate of our statistical uncertainty.
- Free energies can in principle be obtained by adding zero-point-energy (ZPE) and entropy corrections (other than rotational contributions). However, these calculations are extremely expensive for large systems such as those investigated here. Fortunately, the ranking obtained with our energy-based protocol can be used to identify differences in GPBs and PAs between the protomers. Table 4 reports such data for different conformations of free amino acids. The variations in the entropic term (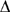
GPB-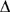
PA) among alternative structures are much smaller (less than 5%) than the differences between the corresponding energy terms (GPB and PA), similar considerations can be done based on literature data [52]. Such differences turn out to be roughly constant, with a dispersion (in terms of standard deviation from the average) smaller than 6kJ/mol. This dispersion is smaller than the error due to the conformational sampling. Furthermore, the estimates of GPB and PA reported in Table 4 and Table 1 in Text S1 fall nicely into the range of experimentally determined values [105]–[109], and fully support the above considerations (vibrational energy corrections to enthalpy and entropy were calculated from harmonic vibrational frequencies. The effect of losing three translational degrees of freedom on going from 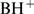 to B (or from AH to A) was also taken into account [103]).

**Table 4:** Thermodynamic data for the protonation reaction of the side chain of some amino acids.

	Folded			Linear			Linear-Folded		
	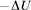	PA	GPB	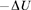	PA	GPB	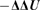	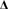 (PA)	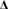 (GPB)
Lys	1024	985	978	961	931	925	−63	−54	−53
Arg	1080	1026	1026	1046	1008	1015	−34	−18	−11
His	1013	983	971	985	953	951	−28	−30	−20
Gln	948	919	919	913	877	872	−35	−42	−47
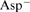	1365	1330	1328	1426	1389	1388	61	59	60
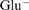	1368	1358	1356	1453	1412	1420	85	54	64

Internal energy change (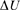), proton affinity (PA), and gas-phase basicity (GPB) values (in kJ/mol) are reported for two limiting situations: (*i*) protonated and deprotonated species in their lowest-energy conformer (labeled as “folded”), and (*ii*) in an extended, all-trans conformer (labeled as “linear”). Differences between these quantities calculated for the folded and linear conformations are also given.

We therefore conclude that the ranking obtained with our protocol provides a reliable identification of the most stable protomers.

### Structural features

We discuss here the salient structural data of the low-energy protomers identified with the protocol outlined above for each system and for every charge state that features, according to our choice of ionizable residues, more than one protomer. More details and additional observations can be found in Text S1. Structural data for each protomer of the considered charge states are reported in Tables 1, 2, and 3.

#### Bradykinin

Four protonation sites are possible: 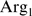, 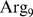, N- and C-terminus. All of the identified lowest-energy protomers from neutral to doubly positively charged states are zwitterions (see Table 1). All of them feature an extensive H-bonding network, and a 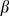-turn-like motif formed by the residues 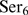-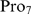-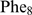-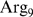, in agreement with Ref. [62]. However, the 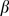-turn does not always feature an 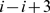 H-bond. The C-term protonation site forms salt-bridges with the others if the geometry allows to do so (see Table 1).

Neutral BK. The neutral form features two lowest-energy protomers, with (*i*) deprotonated C-ter and protonated 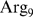 (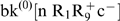) and (*ii*) deprotonated C-ter and protonated 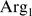 (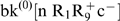)


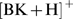
. As for 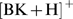, the lowest-energy protomer, 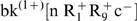, has two Arg residues protonated and the C-ter deprotonated (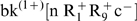). The three charged sites cluster together forming two salt-bridges. The result is consistent with previous *ab initio* calculations [62], [63] and experimental evidences [16], [64].


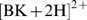
. The lowest-energy protomer of 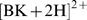 is 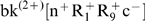. The charged residues form a three-salt–bridge cluster where the C-ter is surrounded by the three positive groups (N-ter, 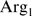, and 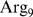). A similar lowest-energy zwitterionic structure for 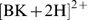 is predicted also by other theoretical studies [62], [110]. From an experimental point of view, no consensus is reached about the zwitterionic nature of 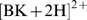. Blackbody infrared radiative dissociation experiments on bradykinin [64] could not rule out the presence of a salt-bridge between the N-terminal arginine and the carboxylate group. On the other hand, 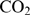 and COOH losses by ultra-violet photodissociation suggest that the zwitterion might not be the predominant species in the gas phase [16].

#### Angiotensin II

Five protonation sites were chosen: N-ter, 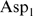, 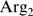, 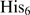 and C-ter. Although the primary sequence of AN is shorter that BK, the presence of an extra acidic residue (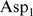) increases the number of possible protomers appreciably. In addition, the existence of two tautomers for neutral His (*i.e.*, 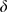 and 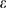 protonated forms) further enlarges the protomer space. We consider here charge states ranging from the monovalent anion to the doubly positively charge cation. All but the latter turn out to be zwitterions (see Table 2).

Lower charge states. The lowest-energy protomer of 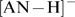 is 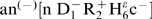. It features the highest number of ionized residues with a deprotonated C-ter and 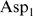 and a protonated 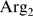. 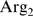 forms two salt-bridges, with both C-ter and 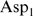. The most stable protomer of the neutral state is 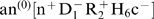, thus highly zwitterionic, with four salt-bridges. Arg is protonated instead of N-ter and His. The most stable structures of [AN+H]^+^ are 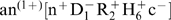, and 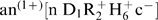, whose relative energies are within 2 kJ/mol. Considering the overall energy ranking, also for this charge state there is a clear tendency to protonate Arg instead of N-ter and His.

The divalent cation. In the case of 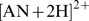, the lowest-energy protomer, 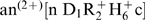, is not a zwitterion and lies 15 kJ/mol below the most stable zwitterion, 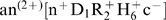.

#### Tryptophan cage

Six protonation sites are possible, N-ter, 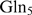, 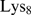, 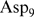, 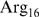, and C-ter, which yield a large number of possible protomers (see Table 3).

For this system, additional 40 ns MD simulations were carried out for the lowest-energy protomers in the gas phase. Comparison is then made with the structural features of the protein in solution. All states considered feature a native-like compact structure (see Table 7 in Text S1), which does not depend appreciably on the charge state. In fact, the gyration radius (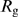) decreases slightly and in a similar way during the dynamics of all of the charge states (see Table 7 in Text S1). This contraction involves the solvent-exposed side chains, which fold onto the protein surface, in agreement with previous MD studies [2], [8], [14], [52]. The side chain rearrangements are such to optimize at best H-bonding and salt-bridges (see Table 3). We remark that, in all of the gas-phase simulations, as a consequence of the absence of water, the Trp residue tends to move away from the hydrophobic core. This result is also consistent with previous investigations [14], [52].

The lowest-energy protomer is a zwitterion for the low charge states (neutral Trp-cage, 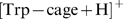, and 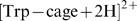). However, the most probable state for 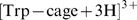 is not a zwitterion.

Lower charge states. The lowest-energy protomers of the neutral state are the zwitterions 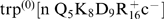 and 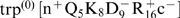 (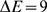 kJ/mol). 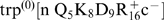 does not feature any salt-bridge, but its ionized residues form a large number of strong H-bonds involving charged groups. In contrast, the highly zwitterionic 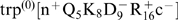 forms four salt-bridges, arranged in a ring structure involving all the charged residues, with a distance between heavy atoms of ionized groups of about 2.85Å.

The lowest-energy protomers of 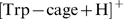 are the zwitterion 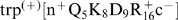, characterized by the N-term/C-term salt-bridge, and the non-zwitterionic species 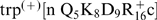 (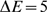 kJ/mol). The presence of a zwitterionic state for 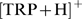 in the gas-phase has been recently proposed by computational and experimental approaches [16], [52]. Patriksson and coworkers [52] identify the zwitterion 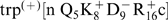 as the most stable protomer, which lies at 41 kJ/mol above the minimum according to our calculations. We found that the most stable protomer differs in having the N-ter charged instead of 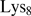 and the C-ter charged instead of 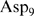. In this regard, we remark that Kjeldsen *et al.* [16] identified the C-ter as the most likely carboxylate of the zwitterion identified by 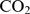 photodissociation experiments on 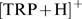.

The 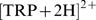 can form 15 protomers. The zwitterion 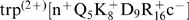 is the lowest-energy protomer. The C-ter forms two salt-bridges with N-ter and 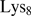. N-ter and C-ter are in very close contact (distance between heavy atoms of 2.6 Å ) and strongly interact via H-bonds, whereas 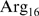 is located at about 3.0Å from the C-ter. This species lies very close to the non-zwitterionic protomer 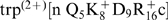 (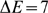 kJ/mol). Zubarev and coworkers [19] suggested a prevalently non-zwitterionic form for 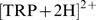 based on photodissociation experiments and on fragmentation patterns by Electron-Capture Dissociation (ECD) of the 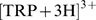 ion. According to these authors, 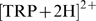 has one positive charge on Arg_16_ and a second positive charge distributed between the N-ter and 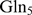. The N-term was found to be the least basic site by ECD [17], [19]. In our results, the protomer with neutral N-term is found only 7 kJ/mol above the lowest-energy protomer with protonated N-term, indicating that this group is likely only partially protonated. 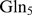 would be unfavorable. Indeed, the lowest-energy species with 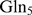 protonated, the zwitterion 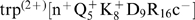, is located at 69 kJ/mol. It will be shown in the next paragraph how our results can provide an alternative explanation of the ECD results.

The trivalent cation. The most probable state for 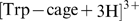 is the non-zwitterion 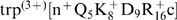. This result is consistent with fluorescence experiments combined with MD calculations carried out by Iavarone *et al.* [25] that led to the identification of the same protomer as the most probable for this charge state. However, our results contrast with the interpretation of recent ECD experiments [19], which points to 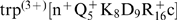 as the most probable protomer. Protonation of 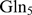 leads to high-energy protomers. The lowest-energy protomer with ionized 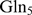, 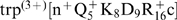, lies at 61 kJ/mol above the minimum.This interpretation was based on the fact that both 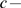 and 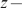
ions generated by ECD show that the 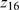 fragment (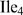-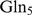 cleavage) exists in two charge states (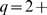 and 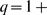, the 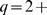 being predominant), whereas the 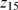 fragment (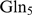-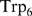 cleavage) is present in the 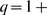 charge state and the 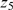 fragment (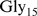-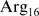 cleavage) in the 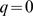 state. No change of charge in the 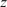 fragments upon 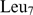-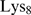 cleavage is observed.

The differences between our and previous findings [19] can be here reconciled considering which interactions are lost upon fragmentation. Visual inspection of the lowest-energy protomer reveals that the charged Lys_8_ side chain is H-bonded to 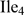 and 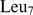 backbone and 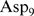 side chain (see Figure 1). In turn, 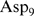 donates a H-bond to 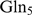. Thus, 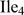, 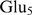, 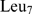 and 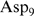 build a H-bond network that internally solvates and, therefore, stabilizes the positive charge on the lysine side chain. Dissociation of 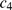 and 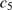 fragments, progressively destroys this network. Such fragmentation could favor spontaneous proton transfer from 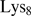 to the departing 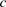 radical ion after the 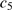 fragmentation. This explanation can also provide a rationale for the anomaly in the charge population of fragment 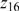.

In conclusion, our results suggest that the observed effect of Gln dissociation might also be explained by its role in charge stabilization and not exclusively by its own ionization in the intact peptide.

### Key stabilizing interactions for peptides and proteins in the gas phase

Our calculations suggest that most of the low-charge states are zwitterions, whilst high charge states might not. We now analyze the key factors for the stabilization of these two different states.

#### Zwitterionic low-charge states

Formation of charge separation between two residues is accompanied by a penalty. To a first approximation, this could be quantified in terms of the intrinsic GPB of the involved residues. The GPB of carboxylates is much higher than that of amino and guanidino groups (see Table 1 in Text S1). Therefore, charge separation between an acid, AH, and a base, B,
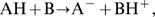
is disfavored in the gas phase by a positive free-energy change 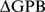 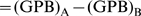 (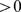), where 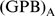 and 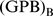 stand for the intrinsic GPB of 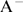 and B, respectively. The larger the 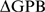, the larger the expected destabilization due to charge separation. In solution, solvation of the charged moieties may counterbalance this energetic penalty [36], [44]. The present results indicate that in the gas phase zwitterions can still be stabilized [3], [35], [38], [39], [51], [63], [111]. Indeed, the propensity of peptides to have a low energy correlates nicely with the number of ionized residues, as well as with 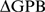
(see Figure 2, first row, see also SI).For each charge state, 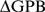 values are relative to the protomer with the lowest charge separation for which 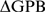 has been set to zero. This is caused by the fact that intramolecular interactions can counterbalance this penalty [3], stabilizing the zwitterionic forms. In fact, such interactions, including (*i*) salt bridges and (*ii*) H-bonds, may reduce differences in the apparent GPB between basic residues and the conjugated base of acidic residues.

- The formation of salt-bridges correlates well with the intrinsic GPB penalty (see Figure 2, last row). A major stabilizing contribution is therefore the formation of favorable electrostatic interactions. As an example, the formation of four salt-bridges in the species 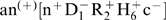 and 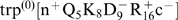 largely counterbalances a very high value of 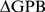 (897 kJ/mol and 842 kJ/mol, respectively). An examination of Tables 1, 2, and 3 suggests that also the salt-bridge geometry is relevant for stabilization. Indeed, consistently with aqueous solution analysis on proteins [112], H-bonded salt-bridges seem to have a major stabilizing effect.

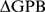
, instead, does not correlate either with the number of ionized H-bonds (see Figure 2, third row), or with the total number of H-bonds (see Figure 2, last row). This observation could be a consequence of the relatively small energetic contribution of H-bonds compared to salt-bridges. However, an important role of H-bonding emerges from the analysis of some specific protomers. For instance, the lowest-energy protomer of neutral Trp-cage, 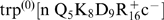, features one charge separation, no salt-bridges and nine ionized H-bonds. The latter therefore must compensate a penalty in intrinsic GPB of 368 kJ/mol. Also informative, in this regard, is the comparison between the fourth and fifth lowest-energy protomers of neutral Trp-cage. Both protomers are located around 25 kJ/mol above the minimum. The former does not present any ionized residues (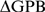 = 0 kJ/mol), whereas the latter, 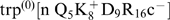, has two ionized residues (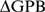 = 416 kJ/mol), which form 6 ionized H-bonds but no salt-bridges. Other similar cases can be found in Tables 1, 2, and 3.

The role of H-bonds is further elucidated by comparing the GPB of single amino acids in their most probable (“folded”) structure and conformations in which the inner H-bonding has been removed, such as the extended, all-trans side chain conformation (We remark that the latter always corresponds to the minimum in the conformational space of alkanes in the gas phase [113]). For basic amino acids, the former is larger than the latter (see Table 4). This fact indicates a greater propensity to acquire a proton when the excess charge can be internally stabilized by H-bonding with the backbone. In acidic residues, instead the former is smaller than the latter, because intramolecular interactions stabilize the negative charge in the folded conformer. Of course, the self-solvation capability of a single isolated amino acid is quite limited. A higher number of H-bond donors and acceptors in peptides and proteins will amplify this effect. An analysis of the structures obtained in this study indicates that each ionized group tends to satisfy at best the same first solvation shell that characterizes the aqueous environment [114], [115] (see Tables 8, 9, and 10 in Text S1). In particular, protonated amino moieties tend to donate three H-bonds (one for each N–H bond) and carboxylates receive four H-bonds in average. The key role of H-bonds is consistent with previous hypotheses [3], [36], [44]. We remark that, as discussed above, H-bonding is only one contribution. The formation of salt bridges provides further (large) energetic stabilization.

#### Non-Zwitterionic high charge states

As the net charge of the molecule increases, more and more basic residues are protonated. Therefore, zwitterionic states imply protonation of residues with progressively lower intrinsic GPB. Consequently, an increasing number of compensating interactions is required. Such compensation might fail because of (*i*) insufficient strength of interactions or (*ii*) topological constraints. As for (*i*), this is clearly the case of Trp-cage at 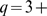, where for the creation of a zwitterionic species it is necessary to protonate residues with low basicity, *e.g.*, 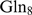, which is not counterbalanced by an adequate internal solvation. As for (*ii*), we remark that the location of protonation sites along the primary sequence considerably influences the possibilities of internal solvation, especially in small unstructured peptides. For instance, the two ionizable side chains in BK (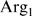 and 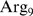) are located at the N- and C-termini, which favor the optimization of the intramolecular interactions for every charge state, because of the flexibility of the peptide backbone. In large proteins, these considerations might be less relevant since the charge-solvation possibilities increase tremendously, and because the folded structure generally offers a favorable environment for ionized side chains.

In summary, as the net charge increases it becomes progressively more difficult to overcome the thermodynamic penalty of charge separation.

## Discussion

A computational protocol aimed at identifying the most stable species of angiotensin II, bradykinin, and tryptophan-cage has been developed and may be easily extended to other systems of similar size. The protocol provides results fully consistent with the experimental data. The results suggest that most of the low-charge states are zwitterions. Intramolecular interactions can stabilize zwitterionic states considerably, by reducing the differences in apparent GPB between basic residues and the conjugated base of acidic residues Based on a combined structural and energetic analysis, we suggest that salt-bridges provide a key energetic stabilization, in agreement with previous findings [3], [38], [51], [63], [116]. Indeed, the stabilization due to salt bridging might be such to reduce enormously the GPB of the biomolecules considered in the present study (up to 900 kJ/mol). H-bonding also has an important role in promoting charge separation. As a result, networks are formed where two (or more) salt bridges are clustered together, whenever it is possible.

Thus, we further corroborate the hypothesis that deprotonated carboxylate groups can be maintained in gas-phase peptide and protein ions produced by electrospray in positive-ion mode (and, vice-versa, protonated basic groups in negative-ion mode) [1], [2], [30], [38], [39], [62], [63], [111]. On the other hand, the formation of zwitterionic species in high charge states requires the protonation of residues with progressively lower GPB, which is accompanied by a large thermodynamic penalty that might not be compensated by internal solvation.

## Supporting Information

Text S1 Supplementary Material. Detailed structural analysis of the protomers and data to support the DFT calculations.
(1.04 MB PDF)
