# Supplementary material for: On the Zwitterionic Nature of Gas-Phase Peptides and Protein Ions
Source: PLoS Comput Biol. 2010 May 6;6(5):e1000775. doi: 10.1371/journal.pcbi.1000775 (PMC2865515; doi:10.1371/journal.pcbi.1000775)
Supplement: Text S1 — Supplementary Material. Detailed structural analysis of the protomers and data to support the DFT calculations. (1.04 MB PDF) [file pcbi.1000775.s001.pdf]

On the zwitterionic nature of gas-phase peptides and protein ions

Roberto Marchese[1], Rita Grandori[2,*], Paolo Carloni[1], Simone Raugei[1,†]
1 SISSA and INFM-DEMOCRITOS center via Beirut 2 - 34151, Trieste, Italy
2 Milano-Bicocca, Piazza della Scienza, 2 - 20126 Milano, Italy
[*] **rita.grandori@unimib.it † E-mail: Corresponding raugei@sissa.it**

Supplemetary Information

Molecular dynamics of Trp-cage in aqueous solution

The NMR structure was solvated with 7000 spc/e water molecules [Berendsen, H. J. C. et al., J. Phys. Chem., 1987, 91, 6269]. A Cl^- ion was added to neutralize the protein charge. After having relaxed the geometry of the system, a constant pressure, constant temperature simulation MD simulation was started at 0K, and the temperature was gradually increased to 300K in 1 ns. The time evolution of the system at 300K was then monitored for 20 ns. The Parrinello-Rahman barostat was employed to control the pressure of the system [Parrinello M. et al., J. Appl. Phys. 1981, 52, 7182] (P = 1 atm). The particle-mesh Ewald method was employed for the calculation of the electrostatic energy. A time step of 1.5 fs was used to integrate the equations of motion. All the hydrogen bond lengths was kept fixed using the the LINCS algorithm [Hess B. et al. , J. Comput. Chem., 1997, 18,1463].

Detailed structural analysis of the protomers

Bradykinin, BK

Neutral BK. For BK ($q = 0$), two close lowest-energy zwitterions were found: (i) deprotonated C-ter and protonated Arg_9 ($bk^{(0)}[nR_1R_9^+c^-]$), and (ii) deprotonated C-ter and protonated Arg_1 ($bk^{(0)}[nR_1^+R_9c^-]$), at 10 kJ/mol above the former. The third possible zwitterionic form ($bk^{(0)}[n^+R_1R_9c^-]$), with protonated N-ter and deprotonated C-ter, is found to be very high in energy (at 53 kJ/mol above the minimum). Extending the study by Rodriquez *et al.* [Rodriquez C. F. et al., J. Phys. Chem. B, 2006, 110, 7528], the protomer with no formal charges was also analyzed. This protomer is 47kJ/mol above the minimum, and it is characterized by the interaction between the two guanidino groups via H-bonding, whereas the the C-ter protonated carboxylic group forms a hydrogen bond with the Arg_1 carbonyl group.

$[BK+H]^+$. With the adopted choice of protonable sites, six protomers are possible for the $[BK+H]^+$ cation. Consistent with previous *ab initio* calculations [Rodriquez C. F. et al., J. Phys. Chem. B, 2006, 110, 7528; Strittmatter E. F. et al., J. Phys. Chem. A, 2000, 104, 6069] and experimental evidences [Kjeldsen F. et al., Chem-Eur J., 2006, 12, 7929; Schnier P. D. et al., J. Am. Chem. Soc., 1996, 118, 7178], the lowest-energy protomer is a zwitterion with the two Arg residues protonated and the C-ter deprotonated ($bk^{(1+)}[nR_1^+R_9^+c^-]$). The other two possible zwitterions ($bk^{(1+)}[n^+R_1R_9^+c^-]$ and $bk^{(1+)}[n^+R_1^+R_9c^-]$) are higher in energy (20 kJ/mol and 73 kJ/mol, respectively). In the two lowest-energy zwitterions, the three charged sites cluster together forming two salt-bridges, while the highest-energy zwitterion features only one salt bridge (between the C-ter and Arg_9), with the N-ter forming three ionized H-bonds (with Pro_2 and Phe_8 carbonyl and Ser_6 hydroxyl group). The three non-zwitterionic protomers, $bk^{(1+)}[nR_1^+R_9c]$, $bk^{(1+)}[nR_1R_9^+c]$, and $bk^{(1+)}[n^+R_1R_9c]$ are located at 20, 91 and 135 kJ/mol from the minimum.

$[BK+2H]^{2+}$. The lowest-energy protomer of the $[BK+2H]^{2+}$ cation is the zwitterion $bk^{(2+)}[n^+R_1^+R_9^+c^-]$. The charged residues form a three-salt–bridge cluster where the C-ter is surrounded by the three positive groups (N-ter, Arg_1, and Arg_9). The remaining protomers, $bk^{(2+)}[n^+R_1R_9^+c]$, $bk^{(2+)}[n^+R_1^+R_9c]$, and $bk^{(2+)}[nR_1^+R_9^+c]$, are much higher in energy (71, 100, and 120 kJ/mol). A similar lowest-energy zwitterionic structure for $[BK+2H]^{2+}$ is predicted also by other theoretical studies [Rodriquez C. F. et al., J. Phys. Chem. B, 2006, 110, 7528; Ewing N. P. et al., J. Mass Spectrom., 2001, 36, 875].

Angiotensin II, AN

$[AN\text{-}H]^-$. The lowest-energy protomer ($an^{(-)}[nD_1^-R_2^+H_6^{\epsilon}c^-]$) features the highest number of ionized residues with a deprotonated C-ter and Asp_1 and a protonated Arg_2. Arg_2 forms two salt-bridges, with

both C-ter and Asp_1. The protomer next in energy (38 kJ/mol) has the same ionized residues, but His_6 is found in the N_δ tautomer, instead of the N_ϵ one. The different tautomeric states lead to different interaction patterns. Indeed, even though the two protomers are nearly identical in terms of distinct types of interactions (see Table 2), a detailed structural analysis reveals a better organization of salt-bridge clustering in $an^{(-)}[nD_1^-R_2^+H_6^\epsilon c^-]$. All of the non-zwitterionic states are very high in energy.

Neutral AN. The lowest-energy protomer of neutral AN, $an^{(0)}[n^+D_1^-R_2^+H_6^\epsilon c^-]$, is highly zwitterionic, with four salt-bridges. Most of the low-energy protomers are zwitterions, too. Table 2 suggests that protonation of Arg side chain is more favorable than protonation of either N-ter or His.

[AN+H]$^+$. For $[AN+H]^+$ three low-energy zwitterions were identified.

[AN+2H]$^{2+}$. In this case, the lowest-energy protomer, $an^{(2+)}[nD_1R_2^+H_6^+c]$, is not zwitterionic. Zwitterionic protomers with deprotonated Asp_1, a residue that contributes to some extent to the charge-state distributions observed in positive-ion mode [Samalikova M et al., J. Mass Spectrom., 2003, 38, 941], are found 43 kJ/mol above the minimum for $[AN+2H]^{2+}$ and 1-2 kJ/mol above the minimum for $[AN+H]^+$.

Tryptophan cage, Trp-cage

Neutral Trp-cage. Two nearly degenerate (ΔE = 9kJ/mol) zwitterions, $trp^{(0)}[nQ_5K_8D_9R_{16}^+c^-]$ and $trp^{(0)}[n^+Q_5K_8D_9^-R_{16}^+c^-]$, were found as the lowest-energy protomers. The zwitterion $trp^{(0)}[nQ_5K_8D_9R_{16}^+c^-]$ does not feature any salt-bridge, but its ionized residues form a large number of strong, charged H-bonds. In contrast, the highly zwitterionic $trp^{(0)}[n^+Q_5K_8D_9^-R_{16}^+c^-]$ forms four salt-bridges, arranged in a ring structure involving all the charged residues, with a distance between heavy atoms of ionized groups of about 2.85Å.

[TRP+H]$^+$. The lowest-energy protomer is the zwitterion $trp^{(+)}[n^+Q_5K_8D_9R_{16}^+c^-]$. The structure of this zwitterion is characterized by a salt bridge between the N- and C-terminal. A non-zwitterionic species, $trp^{(+)}[nQ_5K_8D_9R_{16}^+c]$, is found to be at slightly higher energy (ΔE = 5kJ/mol). The presence of a zwitterionic state for $[TRP+H]^+$ in the gas-phase has been recently proposed by computational and experimental approaches [Kjeldsen F. et al., Chem-Eur J., 2006, 12, 7920; Patriksson A. et al., J. Phys. Chem. B, 2007, 111, 13147]. Patriksson and coworkers [Patriksson A. et al., J. Phys. Chem. B, 2007, 111, 13147] identify the zwitterion $trp^{(+)}[nQ_5K_8^+D_9^-R_{16}^+c]$ as the most stable protomer, which lies at 41 kJ/mol above the minimum according to our calculations. The most stable protomer according to our results differs in having charged N-ter instead Lys_8 and charged C-ter instead of Asp_9. In this regard, we remark that Kjeldsen *et al.* [Kjeldsen F. et al., Chem-Eur J., 2006, 12, 7920] identified the C-ter as the most likely carboxylate of the zwitterion identified by CO_2 photodissociation experiments on $[TRP+H]^+$.

[TRP+2H]$^{2+}$. We identify the zwitterion $trp^{(2+)}[n^+Q_5K_8^+D_9R_{16}^+c^-]$ as the lowest-energy protomer, where the C-ter forms two salt-bridges with N-ter and Lys_8. N-ter and C-ter are in very close contact (distance between heavy atoms of 2.6Å) and strongly interact via H-bonds, whereas Arg_{16} is located at about 3.0Å from C-ter. This species lies very close to the non-zwitterionic protomer $trp^{(2+)}[nQ_5K_8^+D_9R_{16}^+c]$ (7 kJ/mol). Zubarev and coworkers [Adams C. M. et al., J. Am. Soc. Mass Spectrom., 2004, 15, 1087] suggested a prevalently non-zwitterionic state for $[TRP+2H]^{2+}$, based on photodissociation experiments and on fragmentation patterns by Electron-Capture Dissociation (ECD). Such a conclusion would not be in sharp contrast with our results, but the charge location within the most probable, non-zwitterion species is different. The protomer proposed by Zubarev has one positive charge on Arg_{16} and the second

positive charge distributed between the N-terminus and Gln_5. According to our calculations, instead, protonation at Gln_5 would be unfavorable. The lowest-energy species with Gln_5 protonated, the zwitterion $trp^{(2+)}[n^+Q_5^+K_8^+D_9R_{16}c^-]$, is located at 61 kJ/mol.

Energy/structure analysis

Let us start commenting on BK, which presents a small protomer space. For all of the BK charge states considered here, the lowest-energy protomers are zwitterions, which should be largely destabilized with respect to non-zwitterionic states according to the ΔGPB values. For instance, the two lowest-energy protomers of neutral BK, $bk^{(0)}[nR_1^+R_9c^-]$ and $bk^{(0)}[nR_1R_9^+c^-]$, which are close in energy ($\Delta E = 10$ kJ/mol), present a ΔGPB of 368 kJ/mol. Instead, the only possible non-zwitterionic protomer, which is the most favored one in terms of intrinsic ΔGPB, is the highest in energy, being 57 kJ/mol above the minimum. The highest energy zwitterionic protomer, $bk^{(0)}[n^+R_1R_9c^-]$, although largely penalized by a ΔGPB = 540 kJ/mol, is only 49 kJ/mol above the minimum, and 9kJ/mol below the non-zwitterionic protomer. Remarkably, the lowest-energy protomer of $[BK{+}2H]^{2+}$, the zwitterion $bk^{(2+)}[n^+R_1^+R_9^+c^-]$, shows the largest ΔGPB destabilization (540 kJ/mol) for that charge state. The protomer next in energy, the non-zwitterionic species $bk^{(2+)}[nR_1^+R_9^+c]$, which is the most favored protomer in terms of ΔGPB, is 77 kJ/mol above the minimum.

It is evident from this data that the onset of intermolecular interactions considerably reduces the differences in the apparent GPB or PA between basic residues and the conjugated base of acidic residues, eventually inverting the ranking. These considerations can be extended to the other two peptides. For instance, in neutral AN (see Table 2), the non-zwitterionic protomer $an^{(0)}[nD_1R_2H_6^{\delta}c]$ (ΔGPB = 0 kJ/mol) is ranked ninth in energy ($\Delta E = 69$ kJ/mol), while the lowest-energy protomer, the zwitterion $an^{(0)}[nD_1^-R_2^+H_6^+c^-]$ has ΔGPB = 725 kJ/mol. The first three lowest-energy protomers of $[AN{+}H]^+$ are zwitterions with similar energy. Among them, the zwitterion $an^{(+)}[n^+D_1^-R_2^+H_6^+c^-]$ ($\Delta E = 1$ kJ/mol) is close in energy to the non-zwitterionic species $an^{(+)}[nD_1R_2^+H_6^{\delta}c]$ ($\Delta E = 11$ kJ/mol), in spite of the huge gap in ΔGPB existing between the two ions (ΔGPB =897 kJ/mol). Similar astonishing cases are found for other charge states of AN and Trp-cage.

Table 1. Calculated internal energy change (ΔU), proton affinity (PA) and gas-phase basicity (GPB) for the side chains of some amino acids, results compared with previously published experimental and theoretical values.

	BLYP/ GTH/TZV2P			BLYP/ 6-31++G(d,p)	B3LYP/ 6-31++G(d,p) [a]	B3LYP/ aug-cc-pVDZ [b]	Exp.	
	-ΔU	PA	GPB	-ΔU	PA	GPB	PA	GPB
Lys[†]	1024	985	978	1027	998	-	997[a]	-
Arg[†]	1080	1026	1026	1079	1058	-	1052[a]	-
His[†]	1013	983	971	1014	990	-	998[a]	-
$Asp^{-,*}$	1365	1330	1328	-	-	1348	-	1367 [b]
$Glu^{-,*}$	1368	1358	1356	-	-	1353	-	1376 [b]

[a] Dinadayalane, T. C.; Sastry, G. N.; Leszczynski, J. *Int J Quantum Chem* **2006**, *106*, 2920–2933.
[b] Li, Z.; Matus, M. H.; Velazquez, H. A.; Dixon, D. A.; Cassady, C. J. *Int J Mass Spectrom* **2007**, *265*, 213–223.
[†] Structure taken from Ref. *a*.
[*] Structure taken form Ref. *b*.

Table 2. Relative energy of canonical (**c**) and zwitterionic (**z**) arginine conformers (kJ/mol). Conformers are defined according to Ref. *a*.

	**c5**	**c4**	**c3**	**c2**	**c1**	**z1**	**z2**	**z3**
BLYP/GTH/TZV2P	0.0	11.7	2.0	7.2	12.4	9.4	23.8	22.5
B3LYP/6-31++G(d,p) [a]	0.0	8.2	-	7.7	10.6	7.6	17.9	17.1
MP2/6-31++G(d,p) [a]	0.0	3.4	0.8	7.5	13.0	7.1	10.9	15.5
CCSD/6-31++G(d,p) *a*	0.0	5.5	3.3	8.4	8.4	16.7	17.2	19.7

[a] Rak, J.; Skurski, P.; Simons, J.; Gutowski, M. *J Am Chem Soc* **2001**, *123*, 11695–11707.

Table 3. Relative energy (ΔE) in kJ/mol of angiotensin II protomers for the charge state $q = 1+$. BLYP data refers to the energy obtained at BLYP/PP/TZ2P from the computational protocol adopted in this work. B3LYP data refers to all-electrons single-point energy evaluations at DFT/B3LYP level performed on top of BLYP/PP/TZ2P geometries.

N-ter	D_1	R_2	H_6	C-ter	ΔE(BLYP)	ΔE(B3LYP)
0	0	+	+	-	0	0
+	-	+	+	-	1	5
0	-	+	+	0	2	3
0	0	+	δ	0	11	15
0	0	+	ϵ	0	25	22
+	-	+	δ	0	41	48
0	0	0	+	0	42	39
+	0	+	ϵ	-	48	44
+	-	+	ϵ	0	48	50
+	0	+	δ	-	62	69
+	0	0	+	-	81	87
+	0	0	δ	0	85	77
+	-	0	+	0	117	128
+	0	0	ϵ	0	122	135

Table 4. Estimate of the dispersion energy for each protomer of Bradykinin. In each row the following information is reported: protonation pattern (first columns); energy difference with respect to the most stable protomer (ΔE in kJ/mol); difference in the dispersion energy of protomers with respect to the lowest-energy protomer (ΔE_{disp} in kJ/mol) calculated according to the OPLS force field.

N-ter	R_1	R_9	C-ter	ΔE	ΔE_{disp}
q=0					
0	+	0	-	0	2
0	0	+	-	10	0
+	0	0	-	49	1
0	0	0	0	57	5
q=1+					
0	+	+	-	0	0
0	+	0	0	46	-1
0	0	+	0	63	-4
+	0	+	-	67	-15
+	+	0	-	112	13
+	0	0	0	152	-16
q=2+					
+	+	+	-	0	0
0	+	+	0	77	0
+	+	0	0	85	-13
+	0	+	0	93	2

Table 5. Estimate of the dispersion energy for each protomer of Angiotensin II. In each row the following information is reported: protonation pattern (first columns); energy difference with respect to the most stable protomer (ΔE in kJ/mol); difference in the dispersion energy of protomers with respect to the lowest-energy protomer (ΔE_{disp} in kJ/mol) calculated according to the OPLS force field.

N-ter	D_1	R_2	H_6	C-ter	ΔE	ΔE_{disp}
			q=1-			
0	-	+	ϵ	-	0	0
0	-	+	δ	-	43	4
0	-	0	+	-	51	4
0	0	0	δ	-	54	4
0	-	0	δ	0	73	-1
+	-	0	δ	-	84	4
0	-	0	ϵ	0	86	-2
0	0	0	ϵ	-	91	-3
+	-	0	ϵ	-	100	-7
			q=0			
0	-	+	+	-	0	0
0	0	+	δ	-	17	6
0	0	+	ϵ	-	21	3
0	0	0	+	-	32	6
0	-	+	δ	0	37	3
+	-	+	ϵ	-	39	0
+	-	+	δ	-	39	1
0	-	+	ϵ	0	42	9
+	0	0	δ	-	64	7
0	0	0	δ	0	69	8
+	0	0	ϵ	-	73	0
0	-	0	+	0	80	-6
+	-	0	+	-	100	8
+	-	0	ϵ	0	104	4
0	0	0	ϵ	0	105	9
+	-	0	δ	0	106	-4
			q=1+			
0	0	+	+	-	0	0
+	-	+	+	-	1	7
0	-	+	+	0	2	-7
0	0	+	δ	0	11	10
0	0	+	ϵ	0	25	-11
+	-	+	δ	0	41	-1
0	0	0	+	0	42	3
+	0	+	ϵ	-	48	14
+	-	+	ϵ	0	48	-2
+	0	+	δ	-	62	15
+	0	0	+	-	81	9
+	0	0	δ	0	85	8
+	-	0	+	0	117	4
+	0	0	ϵ	0	122	3
			q=2+			
0	0	+	+	0	0	0
+	0	+	+	-	15	-6
+	0	+	δ	0	19	1
+	-	+	+	0	43	-13
+	0	0	+	0	64	-9
+	0	+	ϵ	0	95	-4

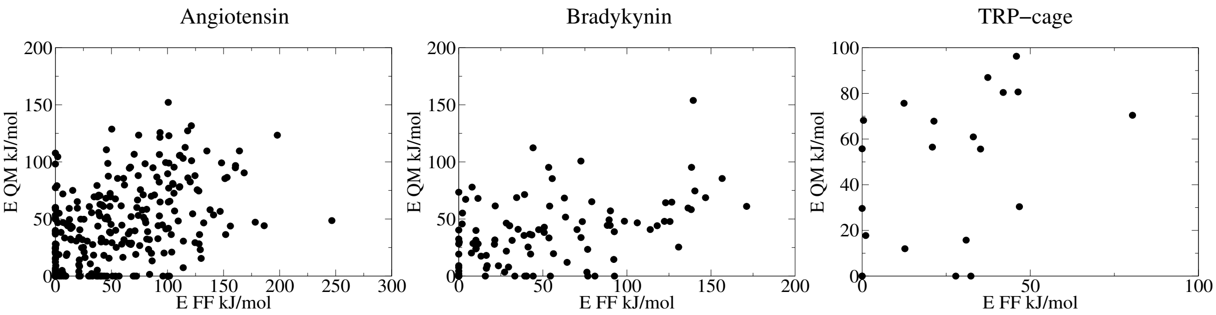

Figure 1. Correlation of relative energies of angiotensin II, bradykinin and Trp-cage conformers calculated at the DFT BLYP/GTH/TZV2P and molecular mechanics OPLS/AA level of theory. Data refer to different protomers and different charge states.

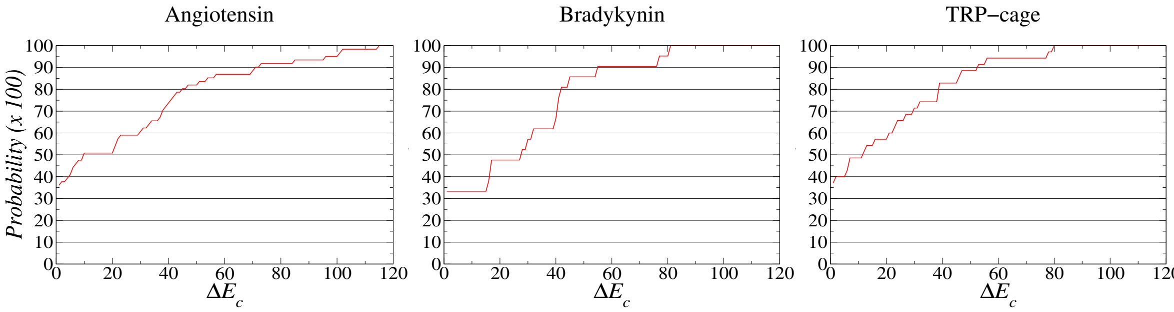

Figure 2. Probability that a DFT BLYP/GTH/TZV2P conformer located within 10 kJ/mol fall more than ΔE_{c} (in kJ/mol) above the OPLS/AA minimum.

Table 6. Estimate of the dispersion energy for each protomer of TRP-Cage. In each row the following information is reported: protonation pattern (first columns); energy difference with respect to the most stable protomer (ΔE in kJ/mol); difference in the dispersion energy of protomers with respect to the lowest-energy protomer (ΔE_{disp} in kJ/mol) calculated according to the OPLS force field.

N-ter	Q_5	K_8	D_9	R_{16}	C-ter	ΔE	ΔE_{disp}
			q=0				
0	0	0	0	+	-	0	0
+	0	0	-	+	-	9	8
0	0	+	-	0	0	20	3
0	0	0	0	0	0	22	0
0	0	+	0	0	-	25	3
0	0	+	-	+	-	33	-1
0	0	0	-	+	0	47	14
+	0	+	-	0	-	95	3
+	0	0	0	0	-	108	13
+	0	0	-	0	0	228	6
			q=1+				
+	0	0	0	+	-	0	0
0	0	0	0	+	0	5	-4
+	0	0	-	+	0	18	4
0	0	+	-	+	0	41	13
+	0	0	0	0	0	66	4
+	0	+	0	0	-	66	-16
+	0	+	-	+	-	72	19
0	0	+	0	+	-	79	3
0	0	+	0	0	0	87	18
+	0	+	-	0	0	99	21
			q=2+				
+	0	+	0	+	-	0	0
0	0	+	0	+	0	7	1
+	0	0	0	+	0	57	2
+	+	+	0	0	-	69	-9
+	+	+	-	+	-	74	-14
+	0	+	-	+	0	104	15
+	0	+	0	0	0	105	9
+	+	0	0	+	-	110	16
0	+	+	0	+	-	116	-4
0	+	0	0	+	0	130	-13
0	+	+	-	+	0	144	12
+	+	+	-	0	0	155	8
0	+	+	0	0	0	132	-21
+	+	0	0	0	0	211	-6
+	+	0	-	+	0	264	9
			q=3+				
+	0	+	0	+	0	0	0
+	+	0	0	+	0	61	-8
+	+	+	0	+	-	62	11
0	+	+	0	+	0	84	-7
+	+	+	-	+	0	113	-4
+	+	+	0	0	0	158	13

Table 7. Statistical data on Trp-cage structure at 300 K in aqueous solution ($q = 1+_{\mathrm{aq}}$) and in gas-phase (radius of gyration: R_g; root mean square displacement with respect to the NMR structure, RMSD)^a.

q	R_{gyr}(Å)	RMSD(Å)
$1+_{\mathrm{aq}}$	6.80(0.08)	1.50(0.23)
0	6.87(0.07)	3.91(0.16)
1+	7.24(0.08)	3.80(0.11)
2+	6.89(0.06)	3.31(0.08)
3+	6.74(0.10)	3.31(0.08)

^{a} Standard deviation from the average given in parentheses.

Table 8. Number of H-bonds formed by ionized residues in bradykinin. The average over all protomers for each charge states and the grand average over all charge states is reported. Standard deviation from the average in parenthesis.

q	N-ter	R_1	R_9	C-ter
0	3.0 (0.0)	4.0 (0.0)	4.0 (0.0)	3.3 (0.5)
1	2.7 (0.5)	3.0 (0.8)	3.3 (0.5)	2.7 (0.5)
2	3.0 (0.0)	2.0 (0.0)	2.7 (0.5)	4.0 (0.0)
	2.9 (0.3)	2.7 (0.9)	3.1 (0.6)	3.1 (0.6)

Table 9. Number of H-bonds formed by ionized residues in angiotensin II. The average over all protomers for each charge states and the grand average over all charge states is reported. Standard deviation from the average in parenthesis.

q	N-ter	D_1	R_2	H_6	C-ter
-1	1.5 (0.5)	3.4 (0.7)	4.5 (0.5)	2.0 (0.0)	2.6 (0.9)
0	2.0 (0.5)	2.8 (0.4)	3.6 (0.5)	2.0 (0.0)	2.8 (0.4)
1	2.1 (0.6)	3.0 (0.6)	3.2 (0.6)	2.0 (0.0)	3.0 (0.6)
2	2.0 (0.6)	3.0 (0.0)	2.0 (0.6)	1.5 (0.5)	4.0 (0.0)
	2.0 (0.6)	3.0 (0.6)	3.2 (0.9)	1.9 (0.3)	2.8 (0.7)

Table 10. Number of H-bonds formed by ionized residues in Trp-cage. The average over all protomers for each charge states and the grand average over all charge states is reported. Standard deviation from the average in parenthesis.

q	N-ter	Q_5	K_8	D_9	R_{16}	C-ter
0	2.5 (0.5)	-	2.5 (0.5)	3.7 (0.5)	4.8 (0.4)	3.3 (0.5)
1	2.5 (0.5)	-	2.7 (0.7)	3.5 (0.9)	4.0 (0.6)	3.2 (0.4)
2	2.8 (0.4)	2.5 (0.5)	2.5 (0.8)	3.8 (0.4)	4.3 (0.6)	3.4 (0.8)
3	2.4 (0.5)	2.4 (0.5)	2.6 (0.5)	4.0 (0.0)	3.6 (0.5)	3.0 (0.0)
	2.6 (0.5)	2.5 (0.5)	2.6 (0.7)	3.7 (0.6)	4.2 (0.7)	3.3 (0.6)
